# Use of implementation logic models in the Quadruple Aim QUERI: conceptualization and evolution

**DOI:** 10.1186/s43058-024-00678-6

**Published:** 2025-01-16

**Authors:** Russell E. Glasgow, Marina S. McCreight, Brianne Morgan, Heidi Sjoberg, Anne Hale, Lexus Ujano-De Motta, Lauren McKown, Rachael Kenney, Heather Gilmartin, Christine D. Jones, Joseph Frank, Borsika A. Rabin, Catherine Battaglia

**Affiliations:** 1 Adult and Child Center for Outcomes Research and Delivery Science, University of Colorado, Anschutz Medical Campus, Aurora, CO USA; 2 Department of Family Medicine, University of Colorado, Anschutz Medical Campus, Aurora, CO USA; 3 Denver-Seattle Center of Innovation for Veteran-Centered and Value-Driven Care, Aurora, CO USA; 4 Rocky Mountain Reginal VA Medical Center, VA Eastern Colorado Health Care System, Veterans Health Administration, United States Department of Veterans Affairs, Denver, CO United States; 5 Department of Health Systems, Management, and Policy, Colorado School of Public Health, University of Colorado, Anschutz Medical Campus, Aurora, CO USA; 6 Division of Hospital Medicine, Department of Medicine, University of Colorado, Anschutz Medical Campus, Aurora, CO USA; 7 Division of General Internal Medicine, Department of Medicine, University of Colorado, Anschutz Medical Campus, Aurora, CO USA; 8 Dissemination and Implementation Science Center, UC San Diego Altman Clinical and Translational Research Institute, UC San Diego, La Jolla, CA USA; 9 Herbert Wertheim School of Public Health and Human Longevity Science, UC San Diego, La Jolla, CA USA

**Keywords:** Implementation strategy, Logic model, Adaptation, QUERI, Pragmatic research, Iteration, Context, Implementation outcomes

## Abstract

**Background:**

Implementation strategies are essential to deliver evidence-based programs that align with local context, resources, priorities, and preferences. However, it is not always clear how specific strategies are selected (vs. others) and strategies are not always operationalized clearly, distinctly, and dynamically. Implementation logic models provide one useful way to conceptualize the role and selection of implementation strategies, plan evaluation of their intended impacts on implementation and effectiveness outcomes, and to communicate key aspects of a project.

**Methods:**

This paper describes our initial plans, experiences, and lessons learned from applying implementation logic models in the Quadruple Aim Quality Enhancement Research Initiative (QUERI) a large multi-study program funded by the Veterans Health Administration (VA). We began with two primary implementation strategies based on our earlier work (i.e., Iterative RE-AIM and Relational Facilitation) that were applied across three different health outcomes studies.

**Results:**

Our implementation strategies evolved over time, and new strategies were added. This evolution and reasons for changes are summarized and illustrated with the resulting logic models, both for the overall Quadruple Aim QUERI and the three specific projects. We found that implementation strategies are often not discrete, and their delivery and adaptation is dynamic and should be guided by emerging data and evolving context. Review of logic models across projects was an efficient and useful approach for understanding similarities and differences across projects.

**Conclusions:**

Implementation logic models are helpful for clarifying key objectives and issues for both study teams and implementation partners. There are challenges in logic model construction and presentation when multiple strategies are employed, and when strategies change over time. We recommend presentation of both original and periodically updated project models and provide recommendations for future use of implementation logic models.

**Supplementary Information:**

The online version contains supplementary material available at 10.1186/s43058-024-00678-6.

Contributions to the literature

- This paper illustrates how implementation logic models can be used to clarify similarities and differences across projects.
- It demonstrates how context and implementation strategies change and adapt over time.
- It discusses nuances and challenges in using logic models to summarize complex interrelated issues.
- It recommends and illustrates the use of multiple logic models to address the points above.

## Background

Implementation strategies are the core of implementation science (IS) [1–3]. They are used to translate evidence into practice. There is a substantial body of work on the definition, delivery, categorization, and reporting of implementation strategies [1, 4–6] Implementation logic models can provide a way for implementation strategies and their intended outcomes to be understood within the context of a project. Logic models have been used for decades by many disciplines to visually depict a project and summarize contextual factors and inputs to a program as well as proximal and distal outcomes [7–10]. In particular, the evaluation and program planning literature has emphasized their use [11, 12]. More recently, implementation scientists have used logic models to organize and guide study design and the implementation, evaluation, and sustainment of IS projects. Implementation teams can use logic models to think through and communicate linkages among context, evidence-based programs, implementation strategies, mediating mechanisms, and outcomes [7, 9, 13–15]. Finally, logic models are helpful to promote collaboration with non-researcher/evaluator partners when the logic model uses terminology that is familiar to those participating in the process.

There are different implementation logic model (ILM) formats [7–9]. These ILMs incorporate the evidence-based program, contextual factors hypothesized to influence implementation and outcomes (i.e., determinants), implementation strategies that address key barriers and facilitators, and implementation and effectiveness outcomes. The determinants and outcomes often come from key constructs in IS theories, models, and frameworks. Some of these logic models are framework agnostic and have been used with different IS frameworks [16–19] and others are specific to a theory or framework.

At present the Implementation Research Logic Model (IRLM) is the most widely used in implementation science [7]. Different variations of the IRLM exist and can be created depending on the study design [7]. The IRLM is designed to be used with different determinant frameworks and outcomes models, but the original publication includes contextual factors from the Consolidated Framework for Implementation Research [17] as determinants, the evidence-based program, implementation strategies, mechanisms through which the implementation strategies are conceptualized to have their impact, and three types of outcomes from the Implementation Outcomes Framework: [19] implementation, service, and client. Adaptations to the IRLM are encouraged to best fit with the needs of the specific project and a logic model tailoring tool is available to support this [7]. Several recent publications have used the IRLM and expanded its application. Czosnek et al [13] used multiple case study methodology to develop a single IRLM. Rodriquez et al [14] conducted multiple improvement cycles and summarized early and mid-project determinants and mechanisms in their IRLM. Finally, Sales et al [15] supported the usefulness of IRLM approaches and called for expansion of it to also address causal mechanisms of determinants in addition to mechanisms of implementation strategies.

We applied an ILM approach in the Quadruple Aim Quality Enhancement Research Initiative (QUERI). The goal of the Quadruple Aim QUERI is to enhance Veteran outcomes and experience, clinician engagement, and reduce cost of care by providing value-based care coordination. The Quadruple Aim QUERI used a similar ILM to the IRLM, but based upon that presented in the Plan section of the Dissemination and Implementation Models in Health webtool [9]. We used the Practical, Robust Implementation and Sustainability Model (PRISM) to characterize determinants (i.e., PRISM multilevel context domains) and its Reach, Effectiveness, Adoption, Implementation, and Maintenance (RE-AIM) implementation outcomes [18, 20].

Using an integrated logic model to organize, contrast, and compare multiple center or portfolio programs such as our Quadruple Aim QUERI allows for connecting projects across the portfolio through identification of shared determinants, implementation strategies, and outcomes while also highlighting differences and the unique operationalization of these elements within individual projects. This highlights cross-project findings and lessons learned while also informing learnings at the individual project level.

The context for the Quadruple Aim QUERI is that Veterans increasingly receive care in both the VA and the community [21, 22]. These dual-use Veterans are at risk for fragmented care coordination across healthcare settings, which contributes to adverse outcomes, experience, and cost of care [23, 24]. The Quadruple Aim QUERI program started in 2020 and aims to transform care coordination in three high-risk populations through implementation of three evidence-based programs:

- The Care Coordination and Management (CCM) project supports implementation, evaluates, and sustains care coordination and integrated case management (CCICM). It is a multidisciplinary model of practice for Veterans with complex or high-risk medical and social needs to improve outcomes, communications, and care engagement [22].
- The Transitions Nurse Program for Home Health Care (TNP-HHC) project is adapted from the VA Transitions Nurse Program [25]. The TNP-HHC is implemented by transitions coordinators (nurse or social worker) to assess social determinants of health and coordinate care for Veterans who require skilled home health care after discharge from a VA hospital.
- The Whole Health Coaching in VA Pain Management Teams (WHCPMT) supports Veterans who suffer with chronic pain and aims to counter the opioid epidemic. WHCPMT centers on holistic and patient-centered care by empowering Veterans to focus on what matters to them.

In this paper we share a set of integrated ILMs that summarize the Quadruple Aim QUERI program. One of our key findings is that due to the dynamic nature of Quadruple Aim programs, the ILMs and their components changed over time. We provide both the original and evolving ILMs for the overall program and for the three component projects. We discuss how we used these ILMs to guide the planning, implementation, evaluation, and sustainment of our portfolio of projects, especially related to applying cross-cutting context assessments, implementation strategies, and outcomes. The discussion also focuses on use of ILMs to depict how the implementation strategies in the Quadruple Aim QUERI changed over time and varied across projects. We conclude with reflections on strengths and limitations of ILMs and suggestions for future directions**.**

## Methods

### Implementation Science (IS) conceptual framework

The overarching IS model guiding the Quadruple Aim QUERI is PRISM. As detailed in Rabin et al [26] and Glasgow et al [27], PRISM is the contextually expanded version of the RE-AIM framework. PRISM focuses on a limited number of multilevel contextual factors (e.g., patients, staff, systems, policies) that are conceptualized to impact the widely used RE-AIM implementation outcomes of Reach, Effectiveness, Adoption, Implementation, and Maintenance [20, 26, 27]. PRISM is a pragmatic model to improve translation of evidence-based programs into health systems (or community) practice and ultimately improve population health [18, 26, 27]. It is a context-oriented IS framework to guide researchers, practitioners, and implementation teams to understand, assess, and address key drivers of outcomes including health inequities, and to address challenges during design, implementation, evaluation, and sustainment of programs. PRISM is a determinant and evaluation framework in the classification suggested by Nilsen [28] and has more recently also been used as a process model to guide implementation adaptations [27].

**Fig. 1:**
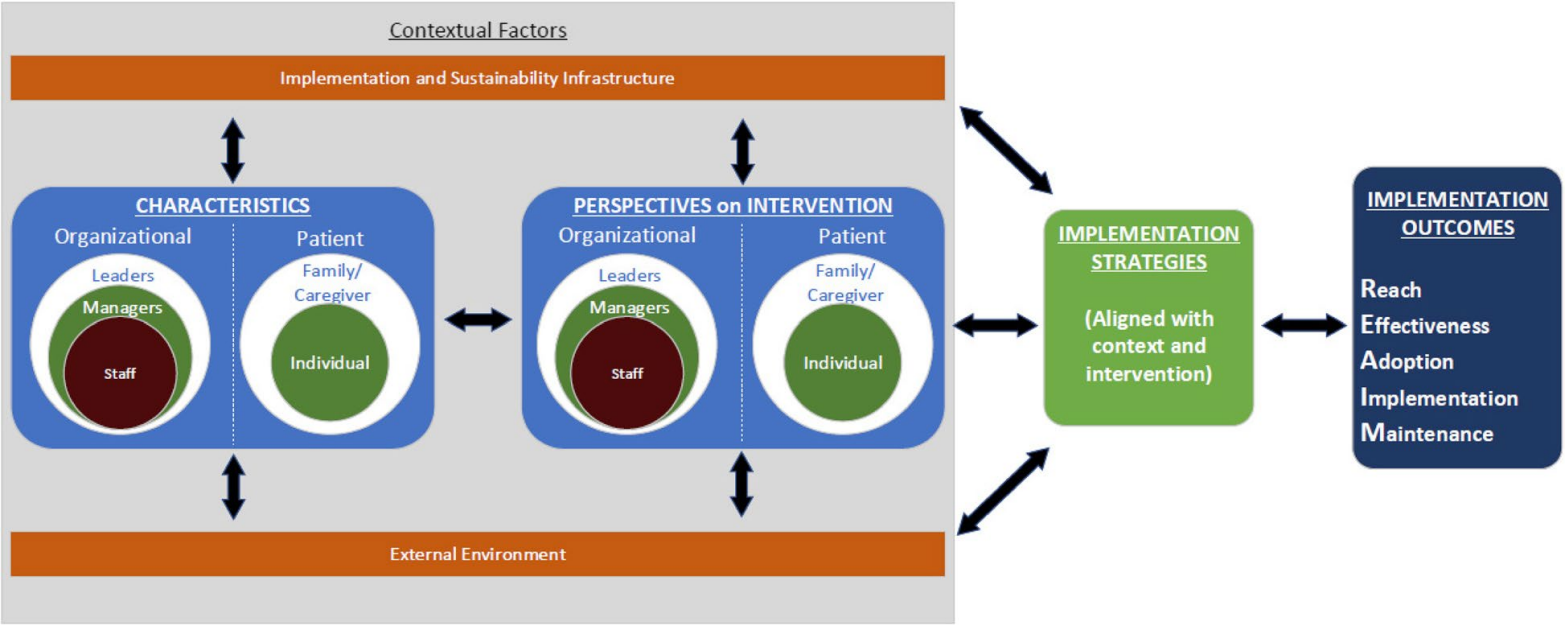
PRISM Logic Model *(from the prismtool.org website with permission)*

As the left hand column of Fig. 1 illustrates, PRISM addresses context by considering how 1) multi-level perspectives (e.g., clinical staff, supervisors, patients) on the evidence-based program; 2) the characteristics of both those delivering (e.g. clinics and clinicians) and receiving a program (e.g., Veteran patients and families); 3) the external environment (e.g. policies, incentives, VA wide mandates); and 4) the implementation and sustainability infrastructure (e.g., resources, workflow, audit and feedback like processes) influence program adoption, implementation, and maintenance. The Quadruple Aim QUERI was designed to incorporate PRISM as an overarching framework that guided planning, implementation, and evaluation across all three projects. We therefore focused on integrating PRISM into our ILM.

### Development of the ILM for the Quadruple Aim QUERI

This original version of our overall ILM was created by our IS Core team as part of our grant application based on our understanding of the Quadruple Aim QUERI program and its component projects. During the project, we worked with the implementing teams representing each of the three projects to create project specific ILMs that further clarified the determinants, implementation strategies, and outcomes unique to individual projects. Then information from the individual projects was used to further refine the overarching Quadruple Aim QUERI ILM. Finally, in writing this paper, we again worked with project teams to create ‘evolved’ ILMs that summarized key components of context and changes to implementation strategies and outcomes. Changes to implementation strategies and proximal outcomes were assessed via implementation team meetings and adaption tracking by IS Core team. While practitioners implementing the program were consulted and provided information to create the original and evolved ILMs, the actual development and modification of the ILMs was done by IS Core staff.

**Fig. 2:**
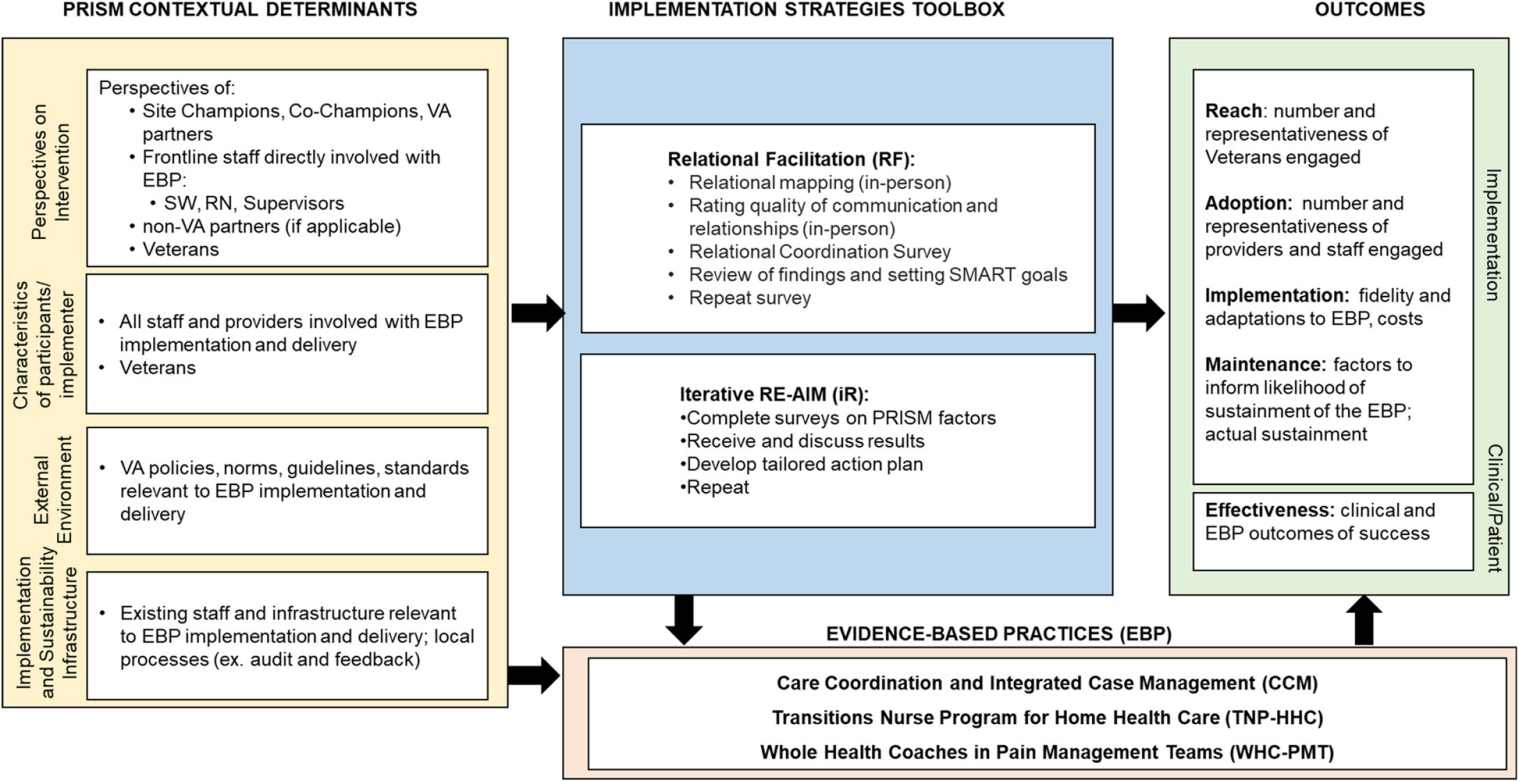
Initial Overarching Logic Model to Evaluate Implementation Strategies

Our application of ILMs differed from most other implementation science use cases in several ways: 1) we used the four core PRISM context categories (see above and Fig. 1); 2) we elected not to include mechanisms in the ILM to decrease complexity and increase accessibility, but listed hypothesized mechanisms as part of our operationalization of the primary implementation strategies (Tables 1 and 3); and 3) we used the RE-AIM outcomes to specify our key implementation and effectiveness.

## Results

Below we present three sets of logic models: (1) the initial overall ILM reflecting how the Quadruple Aim QUERI was proposed and initiated; (2) a revised overall ILM that reflects adaptations that happened during implementation; and (3) three project-specific ILMs reflecting the revised activities and measures for the specific projects.

### Initial overall ILM

We first present and discuss the initial ILM that was developed as part of the Quadruple Aim QUERI grant proposal (see Fig. 2). The left-hand column describes the four key determinant domains from PRISM (i.e., intervention characteristics, recipient characteristics, implementation and sustainability infrastructure, external environment) to capture context of the Quadruple Aim QUERI projects. Initially we proposed to use two primary implementation strategies across all projects: Iterative RE-AIM/PRISM and Relational Facilitation. These primary strategies are described in detail in Table 1using the specifications recommended by Proctor and colleagues [1]. In the next section, we briefly summarize the main activities involved in each of these two strategies and their evolution during the program.

**Table 1:** Operationalization of the Iterative RE-AIM/PRISM and Relational Facilitation primary implementation strategies

Implementation Strategy and Description	Operationalization/Specification
**Relational Facilitation (RF):** Novel methodology grounded in relational coordination (RC) theory to improve relationships and communication skills among and between teams to enhance implementation. Relational mapping, rating quality of communication and relationships, RC Survey followed by review of findings and setting SMART goals	**Actors**: Clinical Leads; iCore team **Actions**: Education, relational mapping, rating quality of RC; RC Surveys, review survey results, SMART goal development with implementors **Targets of Actions**: Site implementation teams **Temporality**: 1–2 months post implementation roll-out; 6–9 months after initial SMART goal setting **Dose**: 2 surveys; monthly/bi-weekly meetings to follow up on SMART goal progress **Justification**: RF improves team dynamics and safety and quality of patient care
**Iterative PRISM (iP):** Using the PRISM conceptual framework to create a novel adaptation of Audit and Feedback that incorporates periodic multi-method assessments to evaluate context and fit of your program during planning, implementation, and sustainment in real-world settings	**Actors**: iCore team; Clinical Leads **Actions**: introduction about the process, iP survey completed by implementors, review of survey results and SMART goal/action planning meeting with implementors **Targets of Actions**: site implementation teams **Temporality**: 6–9 months after implementation starts; repeat 6–9 months after SMART goal setting **Dose**: iterative; # of cycles vary based on funding and project timeline **Justification**: on-going iterative adjustment to the context improves implementation, sustained use, and impact of the intervention

### Iterative RE-AIM

Iterative RE-AIM is an implementation strategy bundle that combines implementation strategies of education, facilitation, audit and feedback, group reflection, and goal setting strategies that are integrated and sequenced using the five RE-AIM dimensions. It is used over time: initially during the planning stage to estimate the impact of an evidence-based program and implementation strategy(ies); during the active implementation stage (once or several times depending on the project, time available, and how quickly results change); and then at the beginning of sustainment to revisit and plan for strategies that are feasible to sustain long-term. Iterative RE-AIM [29, 30] is a conceptually and data-based strategy bundle: 1) it uses the various conceptual categories of the RE-AIM framework to estimate or assess impact on key outcomes (e.g., reach, adoption); and 2) it uses both quantitative and qualitative data to assess impact/progress on the various RE-AIM outcomes at that point in time.

As described by Glasgow et al [29, 30], in each cycle as many health care team members as feasible independently complete brief surveys on the estimated or actual impact of the current or planned strategy on each of the five RE-AIM outcomes. The results are collated (without identifying who gave what ratings), summarized, and used for group reflection, discussion, and action planning for the next time period. We recommend that the team select the one or two RE-AIM dimensions on which there is the largest gap between group ratings of importance and progress, and to then develop an action strategy to enhance progress on that (those) outcomes(s). More detail, forms, questions, and examples of Iterative RE-AIM (and PRISM) are contained on www.re-aim.org and in Gomes et al [31] and in Glasgow et al. [27]

### Relational Facilitation

Relational Facilitation is a pragmatic implementation strategy that combines facilitation with the theory of Relational Coordination (RC) [32] to assess and improve communication and relationships within and between groups [33]. Relational Facilitation was designed to address employee engagement and well-being, critical aspects of the Quadruple Aim QUERI. Facilitation is provided by members of the Quadruple Aim QUERI team and is guided by seven elements of communication and relationships that RC asserts are crucial for enhancing group dynamics, care quality and equity, and to promote innovation adoption and sustainability: frequent, high-quality communication supported by positive relationships with shared goals, shared knowledge, and mutual respect.

The Relational Facilitation process designed for the Quadruple Aim QUERI consisted of multiple steps. Initially, an external facilitator assisted the clinical team in delineating roles and responsibilities necessary for a specific work process (e.g., care coordination). Subsequently, based on these roles, groups constructed a relational map and evaluated RC within and between roles using the RC Survey, a 7-item instrument assessing the seven domains of RC [34]. The survey data underwent descriptive analysis and were shared with the clinical team to inform goal setting aimed at addressing RC deficiencies. Approximately six months later, the RC survey was readministered to gauge changes in RC at the group level over time.

### Implementation outcomes

Key cross-project outcomes in the Quadruple Aim QUERI included proximal RE-AIM implementation outcomes shown in Table 2. These are self-descriptive with two exceptions. First, it is important to emphasize that issues of equity and representativeness of results are important for all RE-AIM outcomes- not just reach [20, 35]. Second, the RE-AIM Implementation dimension includes three components: 1) fidelity or consistency of implementation; 2) adaptations that are made to the strategy (discussed in detail below); and 3) cost, including time and burden to deliver the program or strategy.

**Table 2:** RE-AIM outcomes in Quadruple Aim QUERI

RE-AIM Dimension	Specification
Reach	Percent and representativeness (on demographics, comorbidities, social needs) of those Veterans approached who participated
Effectiveness (primary outcomes)	Varied across projects: Project 1- 30-day readmissions and ED visits Project 2- 30-day readmissions, mortality composite, PCP follow-up Project 3- Veteran and clinician experience; chronic care pain utilization
Adoption	Percent and representativeness of a) clinical settings and b) staff invited that participated
Implementation: -Fidelity -Adaptations -Cost (time and burden)	Consistency of delivery of the key functions (components of the EBP and specific implementation strategies The forms or modifications made to align better with context and emerging data The time required to deliver the EBP and resultant costs
Maintenance (both setting level and patient levels)	The longer term (6–12 months or longer) Veteran outcomes on the primary outcome above The extent to which the sites continued (or adapted) the EBP and strategies after the initial intervention period

### Evolution of the overall and project specific implementation strategies and ILMs

Work operationalizing the strategies and collaborating with the different evidence-based programs and clinical sites on implementation led to adaptations to our original plans and ILM. The impact of the various strategies will be discussed in detail in a separate paper. There were three types of adaptations to our initial implementation plan: (1) expansion of the Iterative RE-AIM process in terms of focus, assessment questions, and implementation interface, (2) modest changes to how Relational Facilitation was delivered, and 3) integration of additional strategies to support the implementation of the evidence-based programs.

### Expansion of Iterative RE-AIM to Iterative PRISM

Implementation of the Iterative RE-AIM strategy revealed that strategies might be better informed and tailored to specific sites if the process also included questions about, and a focus on, alignment of the evidence-based program and implementation strategies with the multilevel implementation context specific to the project clinics, especially their local clinic resources, workflow, and competing demands. We therefore expanded questions and discussion of results on alignment with multilevel context based on the PRISM context domains. The Iterative PRISM strategy bundle, questions, and steps were the same as Iterative RE-AIM, but the process included revised introductory slides, was more interactive, used graphics more prominently, provided additional explanation including clarification of questions, and connection to additional resources on PRISM.

### Adaptations to Relational Facilitation

As reported elsewhere, Relational Facilitation procedures were adapted from the two-day in-person workshops to incorporating the activities into existing virtual meetings with the implementing teams [33]. Specifically, the relational mapping exercise was adapted to accommodate smaller group discussions; and the RC survey was administered during the existing meetings [33].

### Addition of secondary implementation strategies

The other major modification was the addition of other implementation strategies to support the implementation of the evidence-based programs. Some of these strategies were inherent to or part of the intervention. Specifically, all projects included training on the evidence-based program, some level of intervention facilitation, and feedback from qualitative interviews conducted during the planning process or early implementation. These strategies were always planned but were not initially conceptualized as separate implementation strategies, but rather as part of the intervention.

Other strategies were added during implementation to enhance results or address emerging challenges. These strategies included brainwriting premortem [36, 37], collaborative process mapping, site visit feedback, a data dashboard, a patient tracking tool, Electronic Health Records (EHR) note templates, a changing roadmap, a virtual learning collaborative, and feedback (in addition to that received as part of Relational Facilitation and Iterative RE-AIM/PRISM). Specifications of these implementation strategies are listed in Table 3 and as noted there, some of them were only used in a subset of the projects. A particularly interesting implementation strategy added in the CCICM project was a data dashboard based on data from the EHR that provided real time feedback to implementers of the evidence-based program on outcomes to date. This dashboard was interactive and able to conduct queries on several variables to view impact by patient subgroups, etc.

**Table 3:** Operationalization of the additional strategies and activities to enhance implementation

Implementation Strategy/Activity and Description	Operationalization/Specification
**Training:** kick-off meeting with the implementing sites to introduce QUERI team and provide overview of involvement (EBP Projects = all)	**Actors**: Project Coordinators; RN and SW Clinical Leads; iCore; Qual Core **Actions:** introduction about EBP team and process **Targets of Actions:** site implementation teams **Temporality:** pre-implementation **Dose**: Varied from one meeting (1–4 h) to 2-day workshop **Justification:** provide introduction and overview of the involvement of the EBP team
**Brainwriting Premortem:** group exercise to identify site-specific barriers and challenges to consider before CCICM implementation (EBP Projects = CCM)	**Actors**: Project Coordinators; RN and SW Clinical Leads; iCore; Qual Core **Actions:** identify and share pre-implementation barriers and challenges **Targets of Actions:** site implementation teams **Temporality:** pre-implementation **Dose:** one meeting, ~ 30 min, delivered during Training **Justification:** identify pre-implementation barriers to inform CC&ICM implementation
**Facilitation during site meetings:** site meetings to understand context, problem-solve barriers, and discuss ways to enhance facilitators to implementation (EBP Projects = all)	**Actors**: Project Coordinators; RN and SW Clinical Leads **Actions:** Prompt discussion of key implementation issues **Targets of Actions:** site implementation teams **Temporality:** early implementation (weekly) implementation (bi-weekly/monthly) late implementation/sustainment (bi-monthly) **Dose**: on-going meetings (30–60 min) **Justification:** some guidance and prompting needed for successful implementation and successful adaptation
**Pre-Implementation Feedback:** presenting the results of the qualitative inquiry conducted to understand local context, provider and staff perceptions and perspectives (both VA and non-VA) about EBP (EBP Projects = CCM, TNP-HHC)	**Actors**: Qual Core **Actions:** presenting results of the interviews with VA and non-VA partners and stakeholders **Targets of Actions:** site implementation teams **Temporality:** early implementation **Dose**: one meeting (15–30 min to 1 h) **Justification:** understand local context and perceptions about implementing EBP
**Process Maps:** visual representation of workflow and care coordination processes to inform adaptations and costs data collection (EBP Projects = CCM, TNP-HHC)	**Actors**: iCore, Qual Core, RN and SW Clinical Leads **Actions:** design process maps of the current process flow **Targets of Actions:** site implementation teams **Temporality:** pre-implementation, implementation, late implementation **Dose**: iterative **Justification:** to understand the workflow process at the local level and to inform data collection to evaluate adaptations and costs
**Data Dashboard:** visual reports of the outcomes data collected from chart notes completed EBP staff **at** implementing sites (EBP Projects = CCM, TNP-HHC)	**Actors**: RN and SW Clinical Lead **Actions:** overview of EBP elements, demographics, EBP staff contacts **Targets of Actions:** site implementation teams **Temporality:** implementation, presented prior to iP; as requested by implementing site teams **Dose**: iterative (monthly); # of cycles vary based on funding and project timeline **Justification:** to provide real-time snapshot of outcomes to inform the iP assessment
**Site Visit & Feedback:** presenting the results of the qualitative data collection during the site visits conducted to understand local context, provider and staff perceptions and perspectives about EBP and its implementation (EBP Projects = CCM)	**Actors**: Qual Core, RN and SW Clinical Lead **Actions:** present the insights and perspectives learned during the site visits **Targets of Actions:** site implementation teams **Temporality:** implementation **Dose**: one (1–2 days) **Justification:** understand local context and perceptions about implementing EBP

**Fig. 3:**
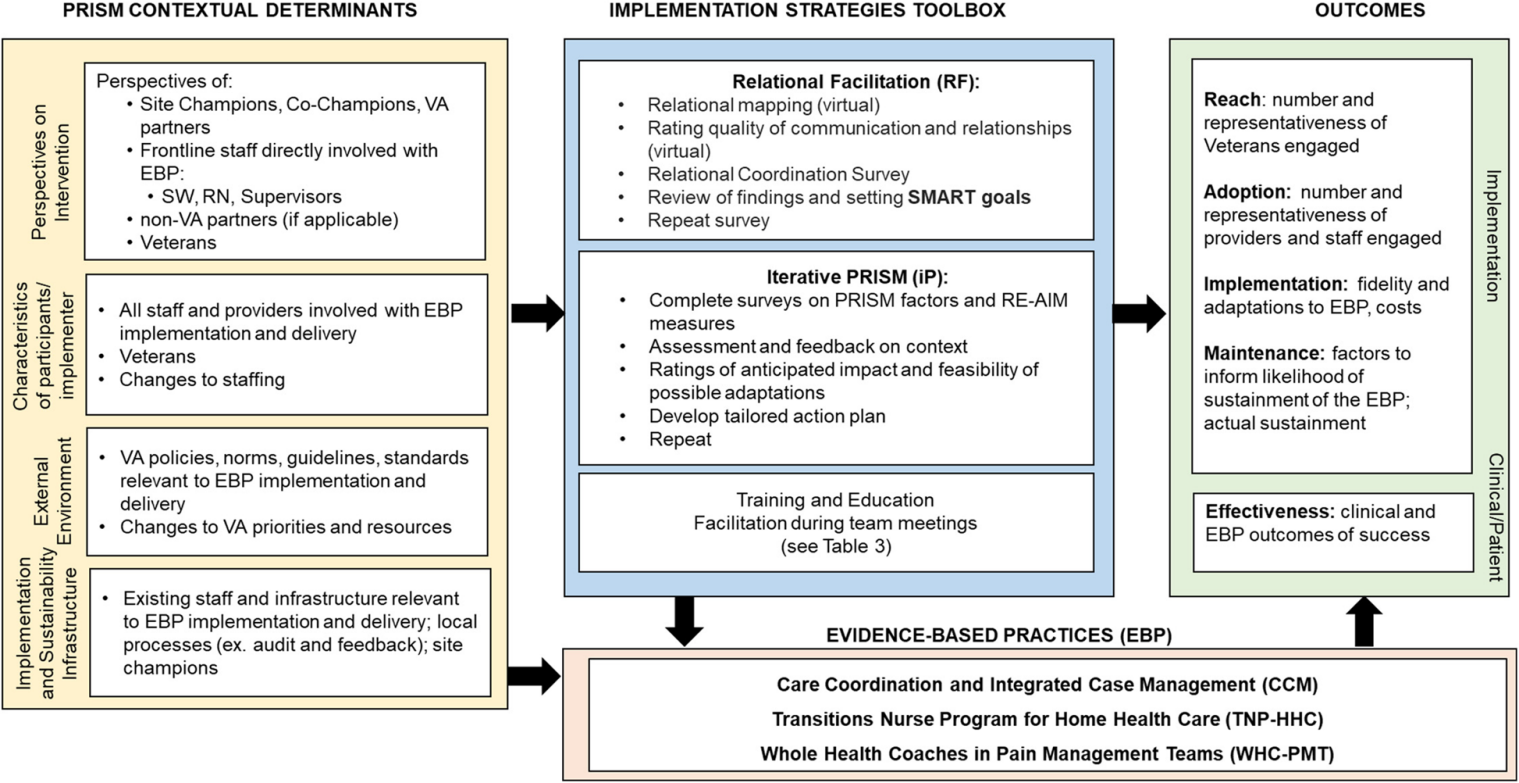
Evolved Logic Model to Evaluate Implementation Strategies

The revised overall lLM in Fig. 3 summarizes the final implementation strategies that evolved, as well as contextual and outcome issues that emerged across all projects.

**Fig. 4:**
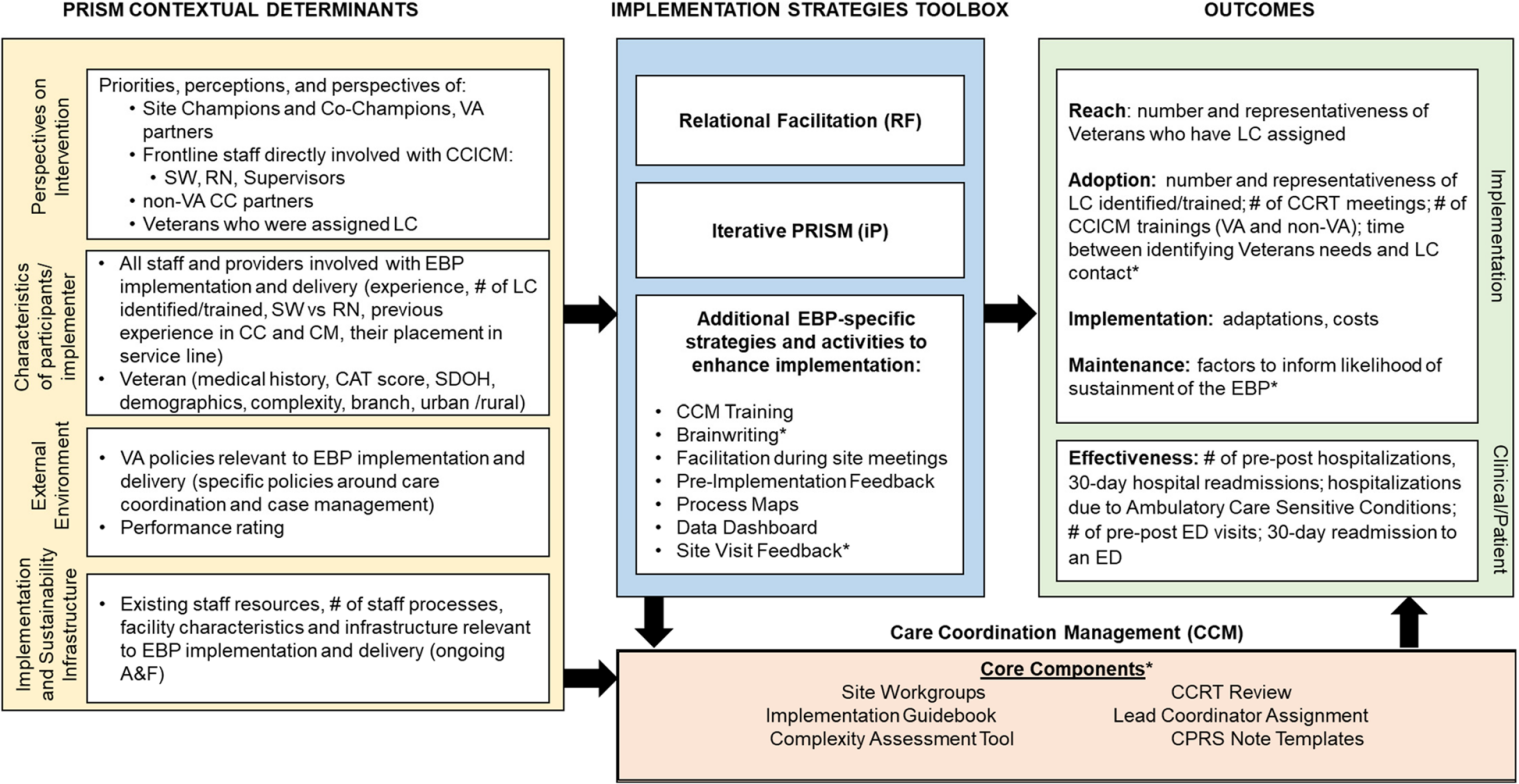
Logic Model to Evaluate Implementation Strategies: Care Coordination Management Project (CCM). *(Asterisk indicates a strategy or component used only in this project and not others)*

**Fig. 5:**
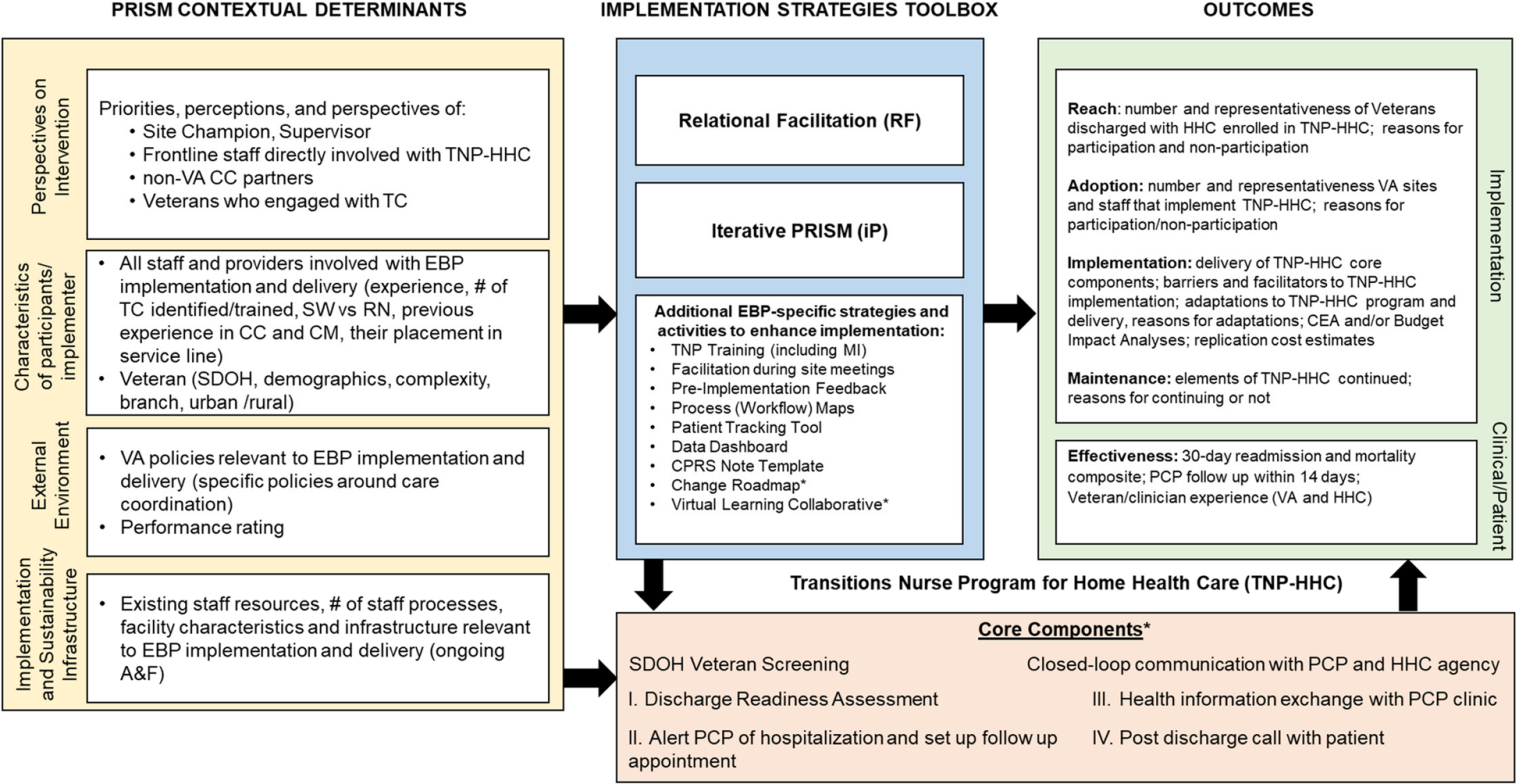
Logic Model to Evaluate Implementation Strategies: Transitions Nurse Program for Home Health Care Project (TNP-HHC). *(Asterisk indicates a strategy or component used only in this project and not others)*

**Fig. 6:**
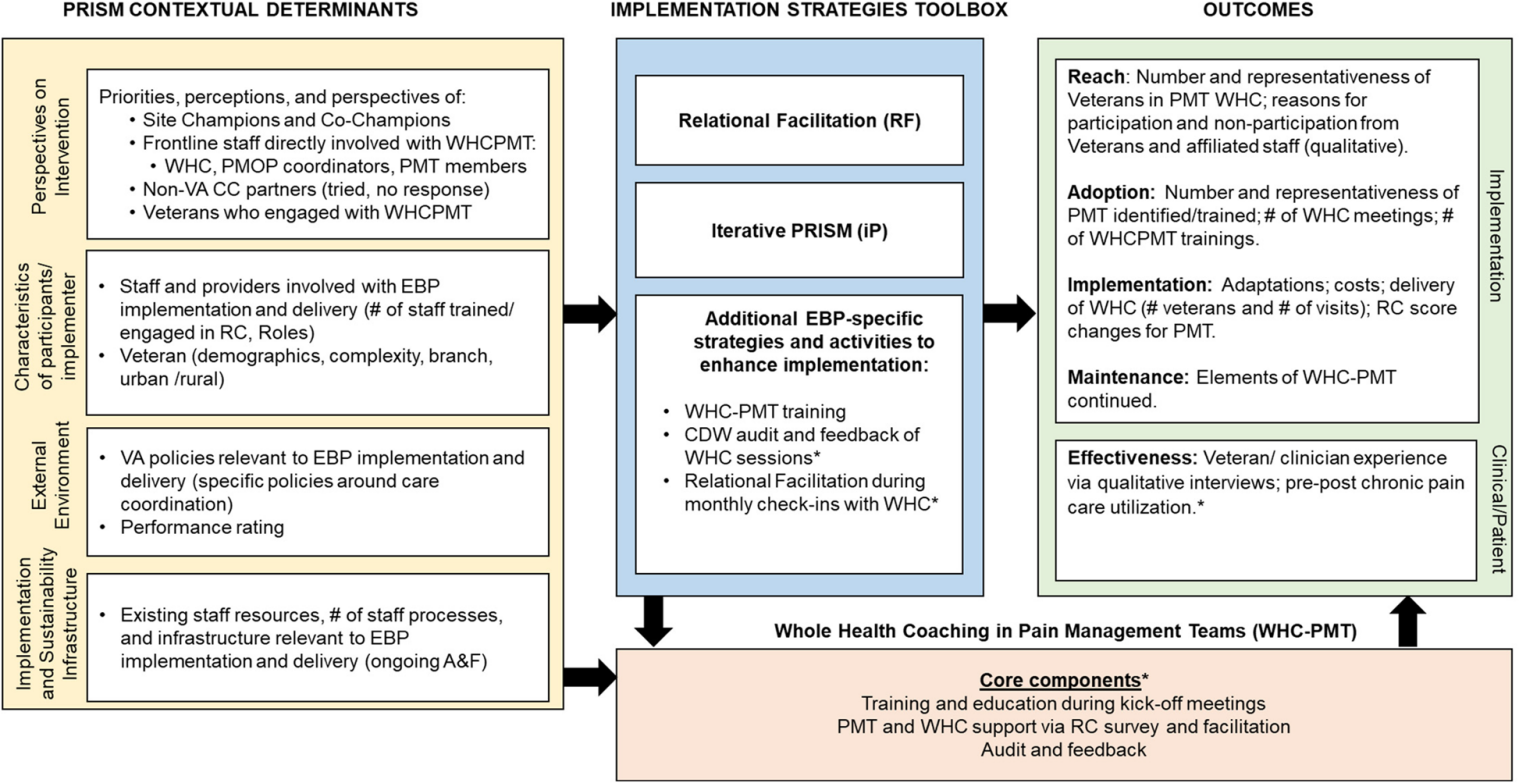
Logic Model to Evaluate Implementation Strategies: Whole Health Coaching in Pain Management Teams Project (WHC-PMT). *(Asterisk indicates a strategy or component used only in this project and not others)*

The implementation strategies used in addition to our two primary ones and the projects in which each was used are summarized according to the Proctor et al. characteristics in Table 3. Figures 4, 5 and 6 display the final lLMs specific to each project listing their specific evidence-based program components, any specific contextual factors, RE-AIM outcomes data, and their specific clinical outcome(s).

As can be seen across Figs. 4, 5 and 6 there were many similarities across projects as you would expect from projects within an integrated program having common overall aims, especially in the categories of contextual factors, several of the implementation strategies, many of the RE-AIM implementation outcomes, and the primary health outcomes (many focused on hospitalizations). Other ways that the project specific ILMs were similar included the key ‘external environment’ contextual factors including national VA operations and national VA policies. All projects also explicitly included multiple perspectives including champions, frontline staff, and Veterans. Implementation strategies that were unique to one project are indicated by an asterisk (*).

The primary ways that the projects differed in addition to their content areas and clinical conditions was in the number and intensity of implementation strategies used. For example, the TNP and CCICM projects employed more strategies than the WHC-PM project, and although not shown in the lLMs, there was also variation across evidence-based programs in the intensity (e.g., dose, time, frequency) of the different strategies, especially the amount of training and facilitation.

## Discussion

A set of overall and project-specific ILMs was developed to support the planning of our initial Quadruple Aim QUERI program proposal; help design and refine the evidence-based programs, implementation strategies, and outcomes; and guide implementation efforts and evaluation. The intervention outcomes and impact of the various implementation strategies will be reported in subsequent papers. The ILMs were helpful in coordinating work across the different Quadruple Aim QUERI projects as well as highlighting both similarities and differences across projects. They were helpful to simplify and visually summarize these complex and dynamic evidence-based programs and implementation strategies, especially as the projects evolved over time.

This report advances the literature on use of ILMs in two ways. First it illustrates how ILMs can be used to summarize 1) changes in program context, activities, and foci over time; and 2) similarities and differences among projects. Second, it presents an integrated set of ILM figures and tables to summarize complex projects. While this is not especially novel, it is to our knowledge the first publication to illustrate this use (e.g., Tables 2 and 3 to elaborate on specifics of implementation strategies too detailed to include in the ILM figure).

The overall and project specific ILMs were found helpful in the following ways:

1. To communicate with operational partners and scientific audiences about the key components and their inclusion across projects;
2. For the implementation team to reflect on progress and fidelity-adaptation issues [38, 39];
3. To support the assessment of which aspects of the PRISM framework, evidence-based programs, and implementation strategies worked best and least well;
4. To understand challenges in sites and the external environment, including new VA mandates, and staff turnover; and
5. To organize and standardize the key measures of implementation success across projects.

What worked less well in our experience with ILMs was conveying the complexity of many of the issues given the limited space. For example, many contextual issues related to characteristics and perspectives of both clinical staff and Veterans were not able to be listed. Similarly, the limited space available to describe implementation outcomes precluded details. It was necessary to create accompanying tables to adequately report characteristics of 1) implementation strategies and 2) RE-AIM outcomes. Another issue is that although we had discussions regarding the mechanisms through which the evidence-based programs and implementation strategies worked, we did not include a column on these in the lLMs for several reasons: there were so many potential mechanisms, we did not specify mechanisms a priori, and many strategies likely operated through numerous mechanisms that this rapidly became overwhelming to report in an ILM. Another departure from the way the IRLM has been used is that we did not indicate linkages between specific contextual determinants and strategies, or strategies to specific outcomes. We discussed this issue but the consensus was that most strategies were related to a large number of determinants and that most strategies related to many outcomes.

Although we started the Quadruple Aim QUERI with two major implementation strategies, Iterative RE-AIM and Relational Facilitation, while conducting the projects, we identified two important issues. The first was that there were some implementation strategies that are inherent in all intervention studies involving training on the evidence-based program and some level of facilitation during the implementation. We had not originally called these out as implementation strategies, but per the definition of implementation strategies (i.e., activities that support the uptake and sustained implementation of evidence-based programs [1]), they qualify as such.

The other finding was that projects added implementation strategies that were not initially planned. Some of these were identified as part of our tracking of adaptations [40, 41] and during discussions with implementation staff. Finally, we also noted that how implementation strategies were operationalized varied across projects and specific clinics/sites and over time. We documented these changes systematically. Similar findings in other implementation research that implementation strategies changed over time led to development of the Longitudinal Implementation Strategy Tracking System to aid the longitudinal documentation and assessment of implementation strategies [6].

We are now using our ILMs to guide our evaluation of the impact of implementation strategies and adaptations, within and across Quadruple Aim QUERI projects. The results of this complex assessment of the outcome of implementation strategies will be reported separately. Finally, we are currently using the ILMs to frame how we prepare for scale-up and sustainment, especially by thinking about how to address contextual factors such as likely major external environment changes, potential staff turnover and reductions, and different workflows.

### Limitations

This report is to our knowledge the first to report both changes in ILMs over time using sequential ILMs and across related projects. We recognize that our application had limitations. Specifically, we did not involve local implementation teams in the initial drafts of the ILMs, and there were challenges in translating some of the IS jargon and concepts to clinical sites and operations partners. At present we do not know the optimal frequency with which to update one’s ILM, the level of expertise needed, the impact of engaging operations partners, or the costs and opportunity costs.

## Conclusion

We found ILMs useful in describing initial project plans by summarizing key presumed contextual factors, implementation strategies, implementation outcomes, and health outcomes. This paper advances the literature on ILMs in the ways as discussed below, and especially in their use to depict *evolution of projects over time and to compare similarities and differences across projects*. Moreover, developing ILMs enhanced our work by helping us to summarize and reflect on changes that occurred during the project implementation.

We recommend further use of ILMs in different types of projects and with different groups of users. To maximize the engagement value of lLMs, we recommend that users 1) specify the initial model in collaboration with site implementation champions (with or possibly without details or discussion of key mechanisms); 2) share a simplified version with implementation teams; and 3) track, record, describe, and summarize changes over time and across projects.

## Supplementary Information

Supplementary Material 1.

## Data Availability

Not applicable.

## Abbreviations

CCM: The Care Coordination and Management project
CCICM: Care Coordination and Integrated Case Management
EHR: Electronic Health Records
ILM: Implementation Logic Model
IRLM: Implementation Research Logic Model
IS: Implementation Science
QUERI: Quality Enhancement Research Initiative
PRISM: Practical, Robust Implementation and Sustainability Model
RC: Relational Coordination
RE-AIM: Reach, Effectiveness, Adoption, Implementation, Maintenance framework
TNP-HHC: The Transitions Nurse Program for Home Health Care project
VA: Veterans Health Administration
WHC-PMT: The Whole Health Coaching in VA Pain Management Teams
